# Mapping Local Dissipation and Entropy Production in Complex and Active Fluids

**DOI:** 10.1021/acs.jpclett.5c02469

**Published:** 2025-10-24

**Authors:** Caroline Desgranges, Jerome Delhommelle

**Affiliations:** † Department of Physics and Applied Physics, University of Massachusetts, Lowell, Massachusetts 01854, United States; ‡ Department of Chemistry, University of Massachusetts, Lowell, Massachusetts 01854, United States

## Abstract

While global entropy production provides a measure of irreversibility, its partitioning into contributions from local regions is key to understanding the mechanisms underlying time-reversal symmetry breaking in complex systems and active matter. Here, by analyzing local heat flows and fluxes, we propose a framework that enables the mapping of local dissipation and entropy production in a nonequilibrium system. We test this approach in simulations of fluids driven through complex environments and active systems. We connect the results across the local and global scales by showing that local dissipation and entropy production satisfy a local version of the usual (global) fluctuation theorem, which accounts for the correlations between the local region and its surroundings. Interestingly, in the case of the active fluid, our analysis reveals that these correlations are of opposite signs for the active (stochastic) and passive (deterministic) contributions to local dissipation.

## Graphical abstract

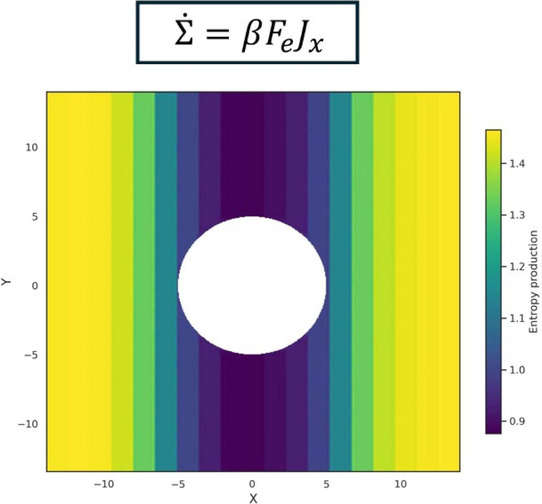

Nonequilibrium and living systems^1,2^ and, more generally, all irreversible thermodynamic processes^3^ are characterized by time-reversal symmetry breaking (TRSB). In such systems, sequences of macrostates and their time reversal can have very different probabilities,^4^ and the ratio of probabilities for time-reversed trajectories is directly related to entropy production.^5–11^ The application of this relation to biological systems,^12–16^ molecular machines,^17–19^ colloidal systems,^20,21^ and quantum systems^22,23^ has opened the door to novel analyses and insights into the behavior of nonequilibrium systems. Recent work has focused on active matter, i.e., systems of bacteria,^24^ synthetic colloids,^25,26^ or even autonomous microrobots,^27^ for which the continuous injection of energy, its transduction into kinetic energy, and the self-propelled motion of active particles lead to TRSB.^28–36^ A complete analysis of the mechanisms underlying TRSB requires, however, the development of methods that provide a measure of local entropy production. Local measures of entropy production have the advantage of identifying regions, or degrees of freedom, where irreversibility is most significant, whereas global measures tend to average out these effects. Furthermore, local entropy production provides access to the amount of work that can be extracted locally and, therefore, leveraged in applications.

The approach introduced in this work builds upon recent efforts to quantify dissipation in complex and active fluids by offering a thermodynamically consistent and spatially resolved framework that links microscopic simulation data to local entropy production measures. The determination of local entropy production in living systems has recently been the focus of intense research. Following the pioneering analysis of broken detailed balance in an active biological system using video microscopy and statistical physics tools,^37^ several novel approaches were developed to evaluate entropy production rates from experimental data. These methods relied on using fluctuating currents and the thermodynamic uncertainty relation,^38^ leveraging estimators based on time asymmetry in waiting times in the absence of currents,^39^ inferring dissipation rates from experimental data using the Fisher information metric,^40^ or developing a mathematical framework to construct estimators that closely approximate the true entropy production rate.^41^ In recent years, machine learning models have also been leveraged to compute probability flows and entropy production using generative modeling^34^ or to determine the mutual information between subsystems using a convolutional neural network.^42^ These approaches complement experimental approaches that assessed the energetic efficiency of living systems by comparing the rate of energy consumption, measured using calorimetry, with the rate of energy dissipation through emergent flows, estimated from microscopy.^43^ Recent advances have also led to a spatial decomposition of entropy production, which was recently performed for the analysis of stochastic field theories,^44^ and to information-theoretic approaches to evaluate local entropy production in simulations and experiments on active matter.^45^ In the latter, configurations of the system are first collected and pixelized to obtain the time evolution of the local density during forward and backward trajectories. In a second step, the information-theoretic measure known as cross-parsing complexity is used to calculate the local entropy production as the difference between the cross-parsing complexity between independently sampled forward and backward trajectories and the cross-parsing complexity between independently sampled forward trajectories. Such an information-theoretic approach is especially appealing when applied to systems for which the governing equations of motion are not known exactly as for biologically active matter.

Understanding how local heat flows and fluxes contribute to dissipation and entropy production in complex systems remains elusive. This is especially crucial in the emerging field of active matter, where energy transduction and dissipation constantly occur at the local scale. In such systems, local heat flows and fluxes develop in a way that differs from the nonequilibrium response of passive systems to external fields and gradients. In particular, having a reliable measure of the local entropy production in these systems would enable the determination of the amount of work that can be extracted locally and used in micro- and nanoscale applications. The interplay among external drive, fluxes, and activity results in a rich collective behavior and complex local structures that require the unraveling of the different contributions to entropy production and dissipation. The extension of the concepts of equilibrium thermodynamics, which include, among others, the definition of entropy through the second law of thermodynamics, to nonequilibrium systems has drawn considerable interest in recent decades. The assumption of local equilibrium allows for the determination of explicit expressions for entropy production as products of generalized forces (affinities, chemical potential gradients, or temperature gradients) by the rates at which irreversible processes (chemical reactions, diffusion, or heat flows) occur.^46^ Although this approach provides a link between entropy production and measurable fluxes and forces, it remains limited to systems that are near equilibrium and where linear relations between flows and forces hold. For far-from-equilibrium systems, recent progress in nonequilibrium statistical mechanics has shown how the theory of dynamical systems leads to a quantitative determination of entropy production.^47^ This theory reveals that, for systems that satisfy the chaotic hypothesis,^6^ entropy production is directly related to phase space contraction and thus to the rate at which dissipative work is performed on the system.^48^ The fluctuation theorem^5,6,11^ and its generalized form, known as the dissipation theorem,^49,50^ provide an expression for the probability distribution of the dissipation, or generalized entropy production, and have been experimentally verified by following the trajectory of a colloidal particle captured in an optical trap.^20,51^ To provide a deeper understanding of the mechanisms underlying TRSB, we build on this approach to map the local dissipation and entropy production in complex and active fluids. With their well-defined sets of equations of motion, particle-based simulations can, in principle, allow for the precise calculation of energy flows, their local partitioning into regions with arbitrarily high resolution, and thus the determination of local dissipation and entropy production in any nonequilibrium system. In fact, dissipation function^11^ Ω can be readily calculated from the equations of motion of the particles as

1 $$\Omega \left(\Gamma \left(t\right)\right)=\beta \frac{dH\left(\Gamma \left(t\right)\right)}{dt}-\Lambda \left(\Gamma \left(t\right)\right)$$

in which H denotes the Hamiltonian, **Γ** = (**q**, **p**) a phase space point (vectors **q** and **p** correspond to the positions and momenta of the particles, respectively), $\beta ={\left(k_{B}T\right)}^{-1}$ the reciprocal temperature, and $\Lambda =\left(\frac{\partial }{\partial \Gamma }\cdot \mathrm{\Gamma ̇}\right)$ the phase space compression factor. However, several open questions have hampered the development of these approaches so far. How can we assess whether a given spatial partition of global dissipation into local contributions provides a physically meaningful measure of local dissipation? Does localization alter the relation between entropy production and the ratio of probabilities for time-reversed trajectories? How does complexity, whether arising from the system’s inhomogeneity or its activity, impact this relation? Furthermore, can such a formalism be extended to existing models for active matter, which generally include both stochastic and deterministic terms in their equations of motion? To address these challenges, we build on the pioneering work of Michel and Searles,^52^ who derived a local version of the dissipation theorem.^49,50^ To establish that the proposed spatial partition of the global dissipation into local contributions provides a physically meaningful measure of local dissipation, we verify that the local contributions satisfy the dissipation and fluctuation theorems,^5,6,11,52^ which was demonstrated using dynamical systems theory and the Sinai–Ruelle–Bowen measures^5,6,47^ and validated experimentally for several colloidal^20,51,53^ and living systems.^12,54^ We add that the fluctuation theorem is applicable to systems subjected to both stochastic and deterministic forces^48,55^ and consider in our analysis examples of deterministic systems for the first two systems studied in this work, as well as a system with both deterministic and stochastic forces in the third system considered in this work. If we consider a local region of size *L* within a homogeneous system and collect over a time interval of duration *t* local dissipation Ω_*L*,*t*_, the local dissipation theorem gives the ratio of the probabilities to observe either a positive dissipation of value *A* or a negative dissipation of value −*A* as

2 $$\ln\left[\frac{p\left(\Omega_{L,t}=A\right)}{p\left(\Omega_{L,t}=-A\right)}\right]=\left(1+\kappa_{L,t}\right)A$$

where κ_*L*,*t*_ captures the correlation between the dissipation in the local region, Ω_*L*,*t*_, and in the rest of the system, $\Omega_{L,t}^{*}$ , as follows.

3 $$\kappa_{L,t}=\frac{\left\langle \Omega_{L,t}^{*}\Omega_{L,t}\right\rangle -\left\langle \Omega_{L,t}^{*}\right\rangle \left\langle \Omega_{L,t}\right\rangle }{\left\langle \Omega_{L,t}^{2}\right\rangle -{\left\langle \Omega_{L,t}\right\rangle }^{2}}$$

These relations provide insight into the local mechanisms underlying TRSB and enable bridging between local and global scales. Indeed, when we consider a trajectory of duration *t* leading to a dissipation of *A*, then the time-reversed trajectory will result in a dissipation of −*A*. When correlations become negligible and κ_*L*,*t*_ = 0, the right-hand side of eq 2 is equal to *A* and we recover the usual statement of the dissipation theorem. Thus, κ_*L*,*t*_ gives a direct measure of the impact of localization, and thus coarse graining, on the relation between dissipation and their probability ratio. Finally, as we will discuss in the cases examined below, when conditions are chosen so that the total energy of the system remains constant ( $\frac{dH}{dt}=0$ ), the dissipation becomes equal to entropy production, thus enabling the determination of the local entropy production through eqs 2 and 3. We now examine how this formalism can be leveraged to map dissipation and entropy production in fluids driven by complex environments and active fluids. We start by considering a fluid driven through a channel of varying width (see the schematic diagram in Figure 1 and Movie M1). The equations of motion for the system particles are given by

4 $$\begin{array}{ll} {\mathrm{q̇}}_{i} & =\frac{p_{i}}{m_{i}}\\ {ṗ}_{i} & =F_{i}+c_{i}F_{e}e_{x}-S_{i}\left[\alpha p_{i}+k\left(q_{i}-q_{0,i}\right)\right] \end{array}$$

in which **q**_*i*_, **p**_*i*_, *m*_*i*_, and *c*_*i*_ = (−1)^*i*^ denote the position, momentum, mass (*m*_*i*_ = 1 for all particles), and color charge of particle *i*, respectively (*c*_*i*_ = 0 for a wall particle), *S*_*i*_ is a parameter equal to 1 for a wall particle and 0 otherwise, **F**_*i*_ is the force exerted on particle *i* by the other particles, *F*_e_ is the intensity of the color field applied along the *x* axis with unit vector **e**_*x*_, α is a Gaussian thermostat multiplier^56^ that keeps the wall temperature constant, and *k* is the spring constant that tethers the wall atom to **q**_0,*i*_, its lattice position.^57^ The color field is akin to an electric field^52,58^ and leads to the onset of a current $J_{x}=\underset{i}{\sum }\frac{c_{i}p_{i}\cdot e_{x}}{m_{i}}$ , with the distinction that the color charges do not interact with each other. *F*_*i*_ arises solely from interparticle interactions, modeled with a short-range Weeks–Chandler–Andersen (WCA) potential.^59^ Throughout this work, σ, *ε*, and *m* define the unit for length, energy, and mass, respectively, and we provide simulation parameters and results in this system of reduced units.^60^

**Figure 1:**
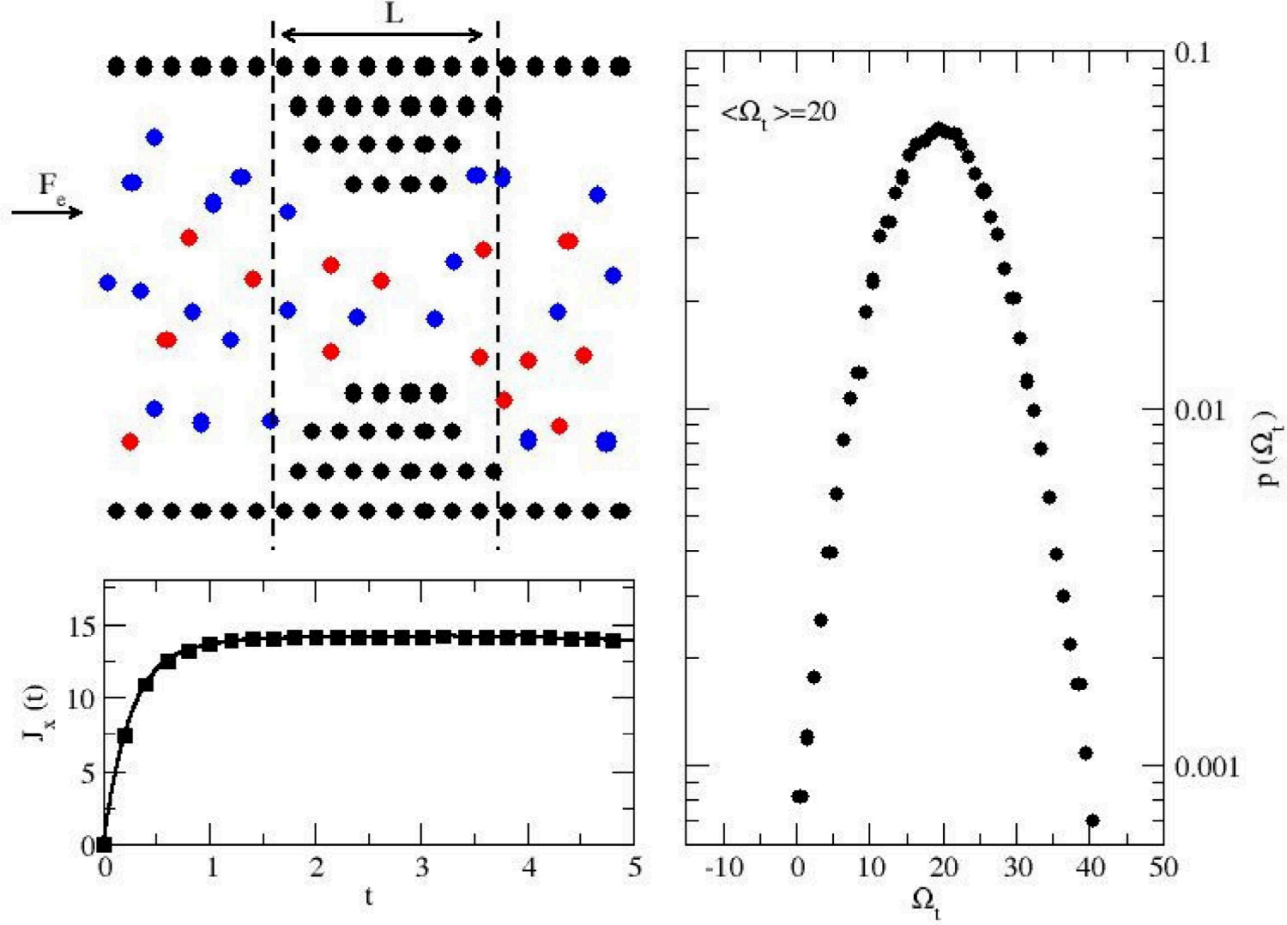
Dissipation in a fluid driven through a channel of varying width. Schematic diagram of the simulation setup (top left). Blue particles have a color charge of *c*_*i*_ = +1 and move with the external field **F**_*e*_ = *F*_e_ **e**_*x*_ (with *F*_e_ = 0.3), while red particles (*c*_*i*_ = −1) move in the opposite direction. Global current for the system averaged over 10^5^ trajectories with different starting configurations at equilibrium (*t* = 0) (bottom left). Distribution for global dissipation Ω_*t*_, collected during the 10^5^ trajectories (*t* = 5) (right).

Local dissipation Ω_*L*,*t*_ (see derivations in the Supporting Information) can be calculated from *J*_*x*,*L*,*t*_, the time-integrated current along the *x* axis over a local region of length *L*, as

5 $$\Omega_{L,t}=\beta F_{e}J_{x,L,t}$$

We first comment on the results obtained for the entire system. The bottom panel of Figure 1 shows the global current, revealing that the system initially at equilibrium (*t* = 0) reaches a steady state over the duration of the nonequilibrium trajectories (*t* = 5). The right panel shows the probability distribution for global dissipation, which corresponds to a Gaussian probability with a mean of $\left\langle \Omega_{t}\right\rangle =20$ and an extremely low probability of observing a trajectory with a negative global dissipation.

Turning to the analysis of local dissipation in Figure 2, we examine how localization impacts local dissipation and the local form of the fluctuation theorem. First, we comment on the results obtained for a bin size of *L* = 4.6, which corresponds roughly to 10% of the entire system, and show in the left panel the distribution for the local dissipation where the channel is narrowest (central bin), where the channel widens (bins next to the central bin), and elsewhere (all other bins). Ω_*L*,*t*_ decreases where the channel is narrowest, i.e., with fewer particles and thus a lower local current (we return to this point in the analysis of the second system). Very interestingly, and unlike for global dissipation, trajectories with negative local dissipation are observed, leading to the finite, non-zero, probability on the left of the plot. The top right panel shows the variation of κ_*L*,*t*_ in the central region, obtained using eq 3, as a function of *L* for 2 < *L* < 50.6. κ_*L*,*t*_ vanishes when the size of the local region increases and takes over the entire system, leading to the usual (global) fluctuation theorem. On the other hand, when *L* → 0, κ increases sharply, indicating the onset of significant correlations between local dissipation (Ω_*L*,*t*_) and dissipation in the rest of the system ( $\Omega_{L,t}^{*}$ ). We also show that the functional fit, proposed in previous work for systems in which all local regions were identical to each other,^52^ generalizes well and accurately models the behavior of κ_*L*,*t*_ in a channel of varying width. We finally examine how localization impacts the ratio of time-reversed probabilities or, in other words, the ratio of the probabilities to observe either positive or negative local dissipation. The simulation results, shown for different *L* values in the bottom right panel of Figure 2, follow the linear relation predicted by the local fluctuation theorem in eq 2. The slope behavior is consistent with eq 2 as it increases when *L* decreases, from a slope close to 1 for large *L* values, and thus low κ_*L*,*t*_ values, to larger slopes when *L* → 0 and large κ_*L*,*t*_ values. The slope is equal to 1.1 for *L* = 10.1 and to 1.5 for *L* = 2, in reasonable agreement with the predicted values of 1 + κ_*L*,*t*_ of 1.13 and 1.82, respectively. The results establish that our approach provides reliable and physically meaningful measures of local dissipation, even when the system is highly inhomogeneous. We show in Figure 3 the spatial distribution of local dissipation throughout the system for a bin width *L* = 4.6. In line with the probability distributions for Ω­(*L*, *t*) shown in Figure 2, we observe that local dissipation reaches a maximum when the channel is the widest, i.e., where the number of particles in the bin that contributes to the local current is the largest. Then, as the channel becomes narrower, local dissipation decreases as the bin contains fewer particles and the local current decreases. Figure 3 thus shows that our approach enables mapping of local dissipation in a fluid flowing through a complex environment.

**Figure 2:**
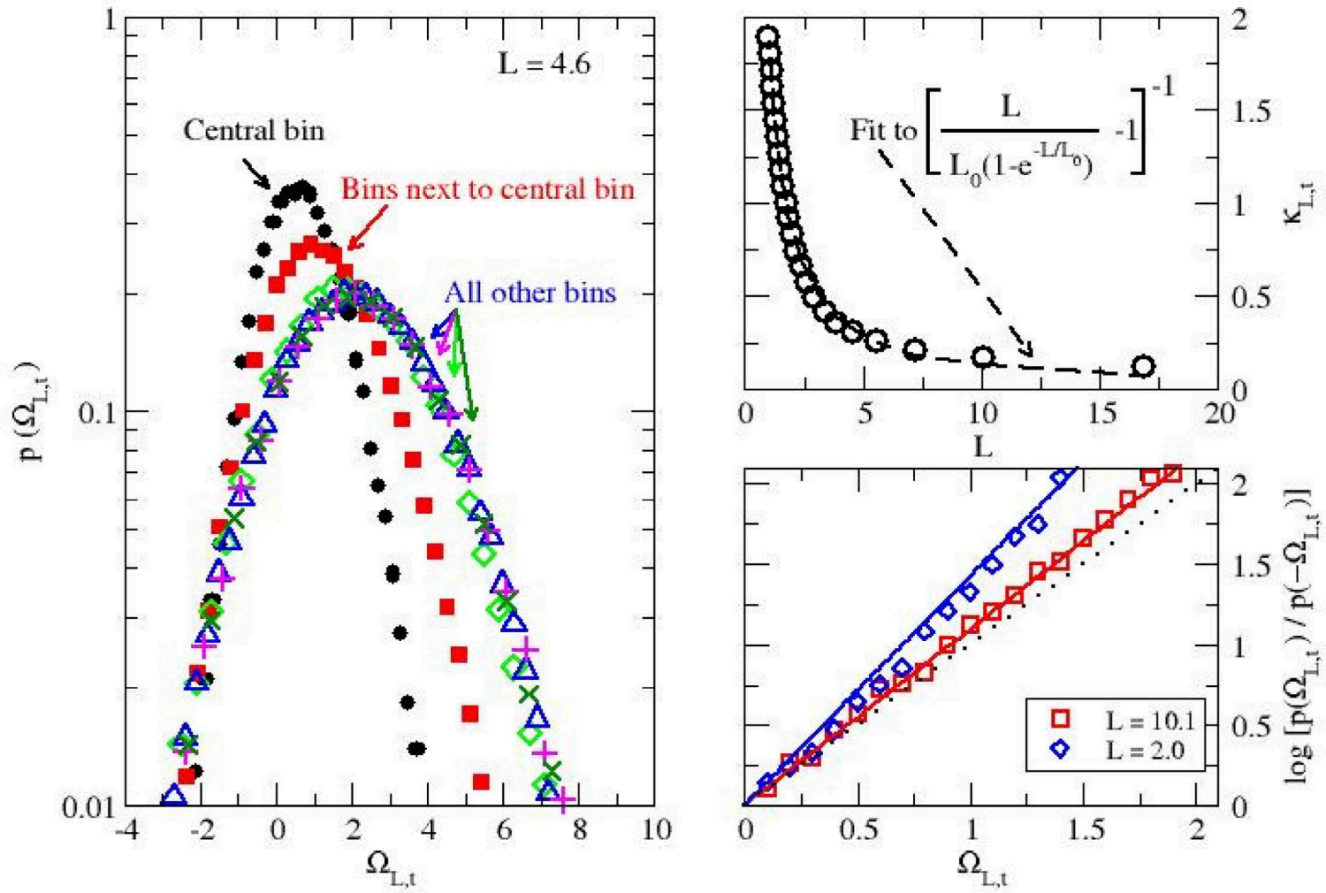
Local dissipation in bins of length *L*, with 2 < *L* < 50.6, for a fluid driven through a channel of varying width. Local dissipation Ω_*L*,*t*_ over bins of length *L* = 4.6 and *t* = 5 (left). The central bin (black), where the channel is narrower, appears on the left and is associated with a greater number of trajectories with Ω_*L*,*t*_ < 0 than for adjacent bins (red) and the remaining bins. *L* dependence of correlation coefficient κ_*L*,*t*_ for ∼20 different *L* values with 2 < *L* < 50.6 (top right). Test of the local fluctuation theorem (bottom right). The plot shows that the simulation data for the left-hand side of eq 2 (empty diamonds for *L* = 2 and empty squares for *L* = 10.1) fall onto lines of increasing slope as *L* decreases.

**Figure 3:**
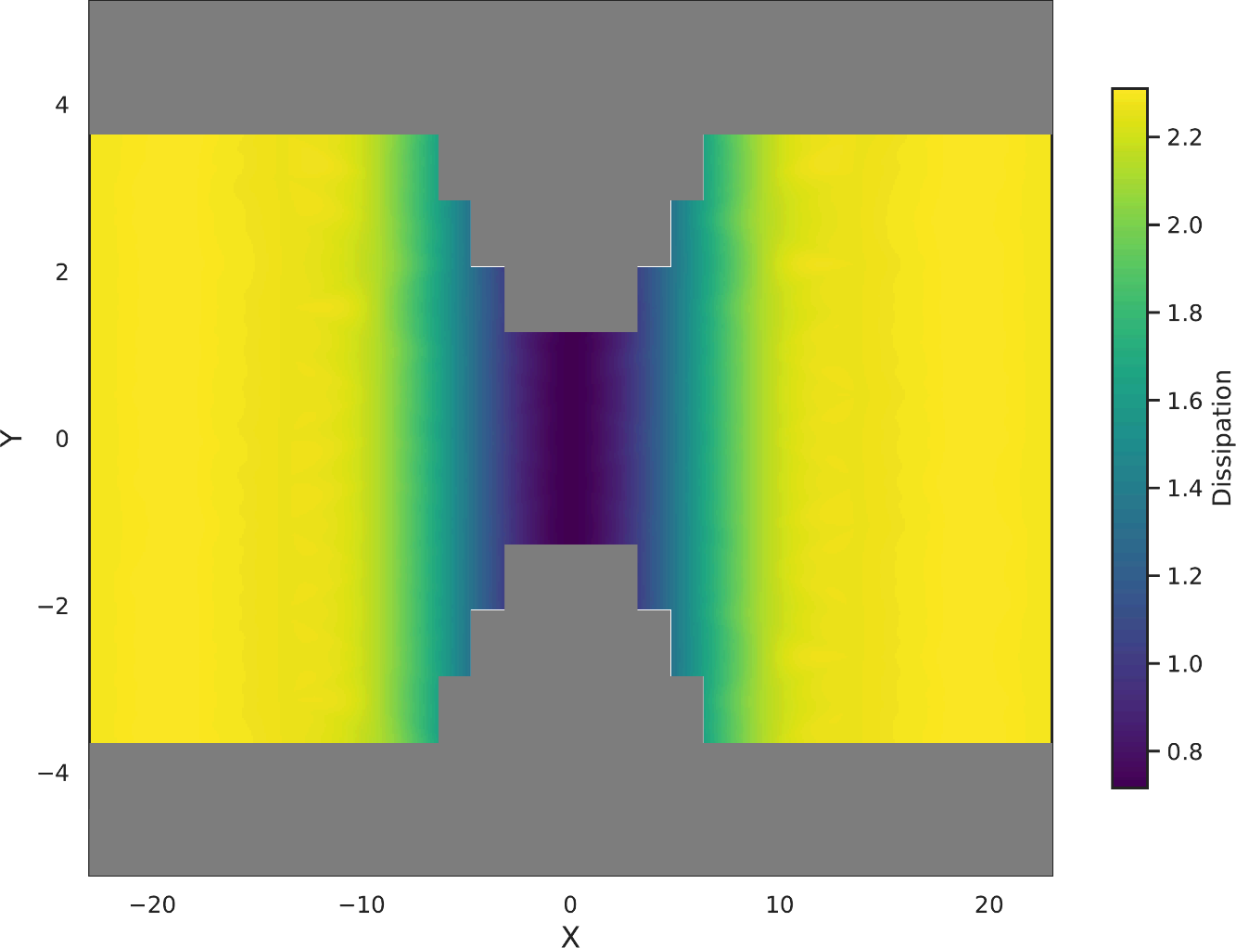
Mapping of local dissipation Ω­(*L*, *t*) for a fluid flowing through a channel of varying width (here, *L* = 4.6 and *t* = 5).

The second system we consider is a fluid driven past a static obstacle (see the schematic diagram in Figure 2 and Movie M2). The equations of motion for the system are

6 $$\begin{array}{ll} {\mathrm{q̇}}_{i} & =\frac{p_{i}}{m_{i}}\\ {ṗ}_{i} & =F_{i}+c_{i}F_{e}e_{x}-\alpha p_{i} \end{array}$$

where the force **F**_*i*_ exerted on particle *i* is the sum of contributions from interparticle interactions and from obstacle–particles interactions.^61^ When α acts as a Gaussian ergostat and keeps the total energy of the fluid constant, we have $\alpha =\frac{\underset{i}{\sum }c_{i}F_{e}p_{x,i}}{\underset{i}{\sum }{p_{i}}^{2}}$ . The deterministic thermostat we use in this study satisfies Gauss’s principle of least constraint, which minimizes phase space compression and preserves time reversibility.^56^ From a mathematical standpoint,^47^ using a Gaussian thermostat leads to a simple phase space dynamics, with the time evolution of the system taking place on a compact manifold, that can be analyzed with a Sinai–Ruelle–Bowen measure using the theory of smooth dynamical systems. Using a Gaussian thermostat thus provides a clear interpretation of dissipation and entropy production arising from external driving forces and a rigorous and efficient framework for studying nonequilibrium steady states and their thermodynamic properties via phase space contraction. The dissipation function then becomes equivalent to Σ̇ , the rate at which entropy is produced,^50^ leading to $\Omega =\mathrm{\Sigma ̇}=\beta F_{e}j\left(t\right)$ (see the Supporting Information for complete derivations). After integration over trajectory duration *t* and the local region of size *L*, we obtain the local entropy production $\Sigma_{L,t}=\int_{0}^{t}{\mathrm{\Sigma ̇}}_{L}\;dt$ reported in Figure 4a–c. Figure 4a shows the probability distribution for Σ_*L*,*t*_ for a region with *L* = 1.5 that is centered on the origin of the disk-shaped obstacle of radius *R* = 5. The local entropy production exhibits a tail in the negative region, highlighting that a fraction of the trajectories leads to negative local entropy production. We then compute the asymmetry function (left-hand side of eq 2) and show the results in the bottom right panel of Figure 4a. The results are accurately modeled by a linear fit, thereby confirming the reliability of our approach for local entropy production, even for very small local regions. To further interpret the results, we plot in Figure 4b the spatial variations across the system of local entropy production, local current *J*_*x*,*L*,*t*_, and local number of particles *n*_*L*,*t*_ across the system. The three plots exhibit a drop around *x* = 0, where the disk-shaped obstacle is located, with Σ_*L*,*t*_, *J*_*x*,*L*,*t*_, and *n*_*L*,*t*_ all decreasing by about 40%. Our approach thus allows for the mapping of how entropy is produced locally as shown in Figure 4c with an arbitrarily high resolution. Very interestingly, the results for the local entropy production, together with those obtained for the first system on local dissipation, reveal that the spatial partition proposed here holds for two very different mechanisms to account for heat dissipation. While we applied a Gaussian ergostat to the fluid for the second system, we used for the first system a realistic model for heat removal since heat was removed through the boundaries. In the first system, the Gaussian thermostat was applied only to the atoms of the nanochannel walls. This means that the thermostat did not act on the fluid atoms and, thus, on the measured local dissipation. Thus, the wall atoms played the role of a heat bath, and the fluid was thermalized through the wall–fluid interactions. Regardless of the scenario chosen for heat removal, the spatial partition of the global dissipation and entropy production so obtained satisfies a fluctuation theorem in both cases. More generally, we add that the choice of a specific type of deterministic thermostat does not impact the simulation results obtained for the dissipation and fluctuation theorem. In previous work, Williams et al.^62^ studied an infinite class of fictitious time-reversible deterministic thermostats and showed that the fluctuation theorem was independent of the precise mathematical details of the thermostating mechanism.

**Figure 4:**
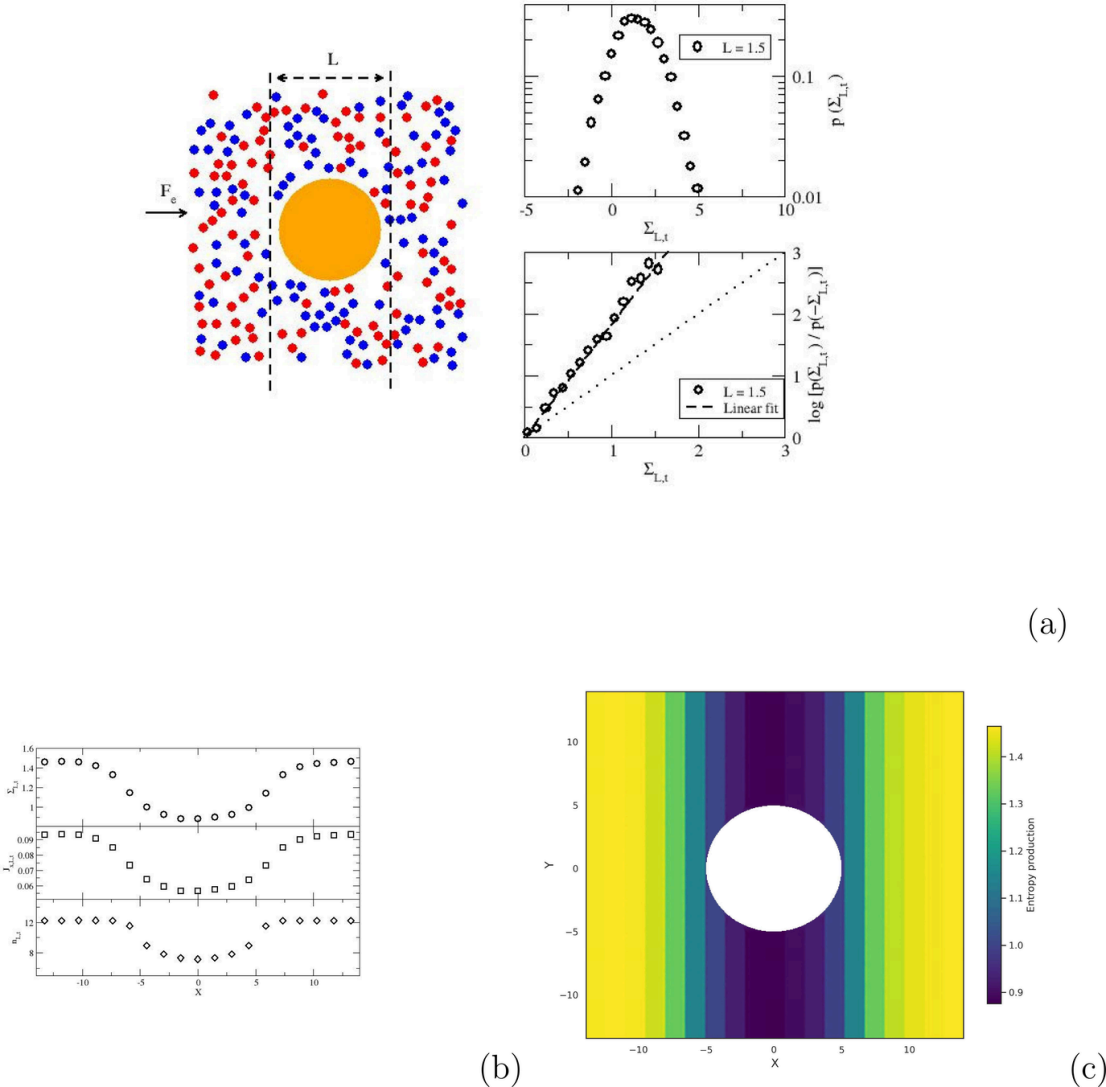
Local entropy production in a fluid driven past a disk-shaped obstacle. (a) Schematic of the system with the obstacle shown in orange (left). Probability distribution for the local entropy production Σ_*L*,*t*_ for *L* = 1.5 and *t* = 5 (top right) and verification of the local fluctuation theorem for Σ_*L*,*t*_ (bottom right). (b) Spatial variation across the system of local entropy production Σ_*L*,*t*_, local current *J*_*x*,*L*,*t*_, and local number of particles *n*_*L*,*t*_. (c) Mapping of the entropy production across the system.

We finally test our approach on an active fluid subjected to a color field (see the schematic diagram in Figure 5 and Movie M3). As in the AOUP model,^63^ we model the activity of a particle by adding an Ornstein–Uhlenbeck process along the *x* axis to the equation of motion for the momentum of each particle. The equations of motion are

7 $$\begin{array}{ll} {\mathrm{q̇}}_{i} & =\frac{p_{i}}{m_{i}}\\ {ṗ}_{i} & =F_{i}+c_{i}F_{e}e_{x}+{\mathrm{\xi ̇}}_{i}e_{x}-\alpha p_{i}\\ {\mathrm{\xi ̇}}_{i} & =-\frac{\xi_{i}}{\tau }+\eta_{i} \end{array}$$

where α denotes a thermostat multiplier defined as $\alpha =\underset{i}{\sum }p_{i}\cdot \left(F_{i}+\left(c_{i}F_{e}+\xi_{i}\right)e_{x}\right)/\underset{i}{\sum }{p_{i}}^{2}$ . η_*i*_ is a Gaussian noise of zero mean and covariance $\left\langle \eta_{i}\left(t\right).\eta_{i}\left(t+s\right)\right\rangle =2\sigma^{2}\delta \left(s\right)/\tau$ . Very interestingly, the dissipation can be written as the sum of a deterministic term ( $\Omega_{F_{e}}$ ) and an active stochastic contribution (Ω_ξ_) as

8 $$\Omega =\Omega_{F_{e}}+\Omega_{\xi }=\beta F_{e}J_{x}+\underset{i}{\sum }\xi_{i}p_{x,i}$$

**Figure 5:**
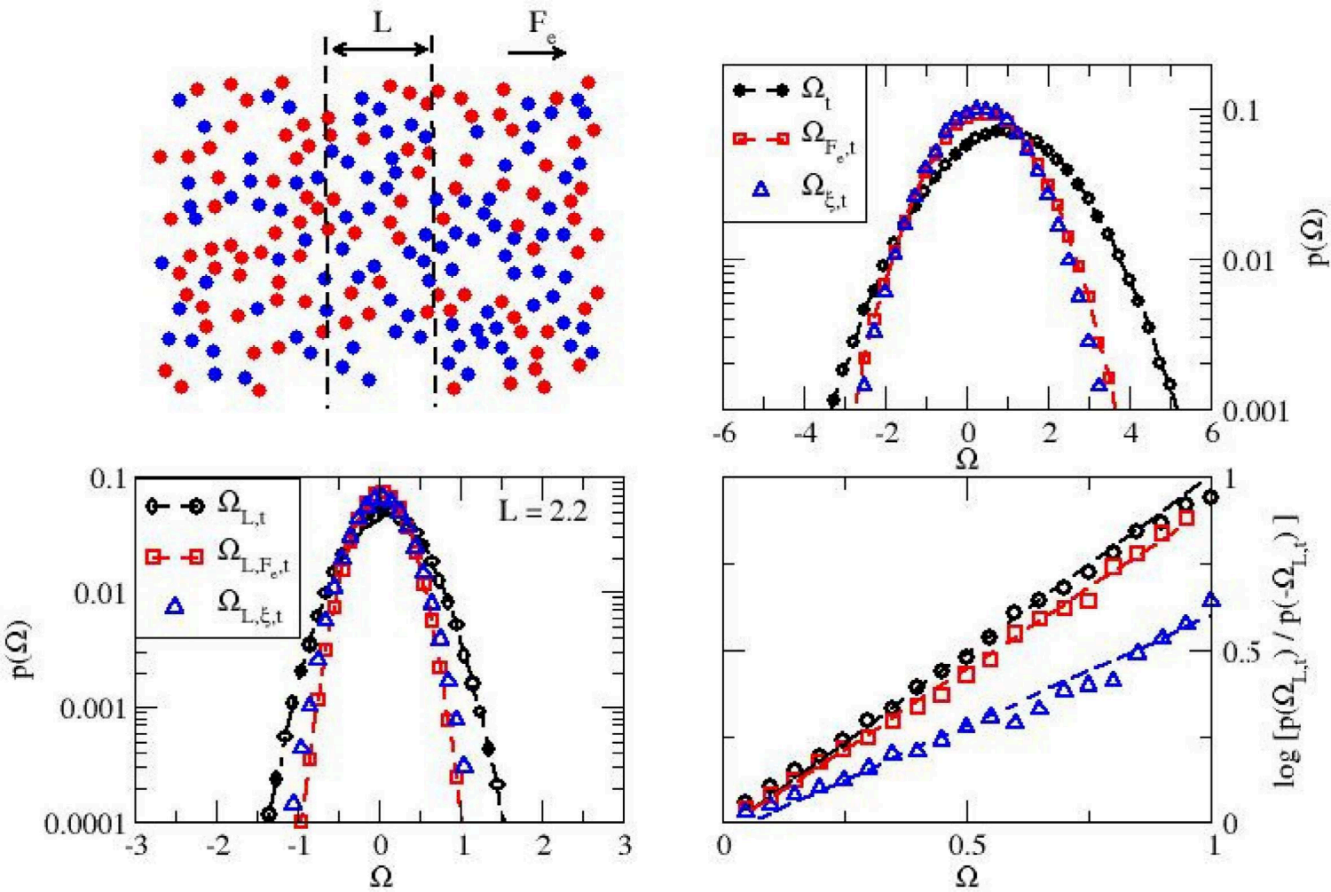
Dissipation in a driven system of active particles. Schematic of the system (top left). Global dissipation (Ω_*t*_) with its deterministic ( $\Omega_{F_{e},t}$ ) and stochastic (Ω_ξ,*t*_) components for *t* = 3 (top right). Local dissipation (Ω_*L*,*t*_) together with the deterministic ( $\Omega_{L,F_{e},t}$ ) and stochastic (Ω_*L*,ξ,*t*_) contributions for *L* = 2.2 (bottom left). Asymmetry functions for the local dissipation and its deterministic and stochastic components (bottom right).

To better evaluate our approach, we ensure that the deterministic term and the active stochastic term contribute similarly to dissipation by setting *F*_e_ = 0.05, σ = 2*F*_e_, and τ = 0.1. The global dissipation is shown in the top right panel of Figure 5, while the local dissipation over a region of width *L* = 2.2, corresponding to roughly a tenth of the overall system, is shown in the bottom left plot. We also include in both plots the corresponding deterministic and stochastic contributions. As expected, we observe that local dissipation and the local contributions are characterized by much narrower probability distributions than for their global counterparts and have mean values that are about an order of magnitude smaller. We turn to the behavior of the asymmetry function for local dissipation in the bottom right plot of Figure 5. Since the field intensity is low, we expect κ_*L*,*t*_ to be close to 0 and the conventional fluctuation theorem to hold. This is confirmed by the measured values for κ_*L*,*t*_ (below 0.25 for *L* ≥ 2.2) and the results shown in Figure 5, with the simulation data for the total local dissipation falling onto a line of slope 1. Very interestingly, the total local dissipation and the deterministic and active stochastic contributions all exhibit a linear asymmetry function. While both the active (stochastic) and the driven (deterministic) terms thus satisfy a local fluctuation theorem, we observe that localization impacts the two contributions. For the active part, we find that the slope for the asymmetry function is below 1 (0.64 ± 0.03), unlike what is observed for the deterministic contribution and for the total local dissipation in the case of this system and for the local dissipation obtained for the first two driven systems studied here. This means that, in the case of the active contribution, the sign of local factor κ­(*L*) becomes negative, which indicates a negative correlation between the local region and its surroundings in marked contrast with the observed behavior for the deterministic contribution and the other deterministic systems. We add that this behavior is consistent with results obtained in previous work for the global dissipation in passive systems driven by a noisy field.^64^

In this work, we introduce a protocol for the determination of local dissipation and entropy production through the analysis of local heat flows and fluxes. While global dissipation and entropy production provide insight into the onset of irreversibility, their partitioning into contributions from local regions is key to understanding the complex mechanisms that take place in inhomogeneous systems and active matter. The systems studied in our work capture key aspects of flows in complex environments and active systems. Living systems often have to navigate through complex environments in biological settings^65–67^ such as, e.g., a fluid flowing in channels of varying width as modeled in the first system examined in this work or a fluid flowing past a large obstacle as modeled in the second system we study. The third system we examine retains key ingredients of minimal active particle models,^26,63,68–70^ which account for motility-induced phase separation^28^ or stimulus-driven pattern formation in bacteria,^24^ and allows us to determine local dissipation in a fluid of active particles modeled as disk-shaped repulsive cores supplemented with active Ornstein–Uhlenbeck processes.^63,70^ We assess the reliability of our approach by mapping the local dissipation and entropy production in fluids driven through complex environments, i.e., through nanochannels of varying width or past a fixed obstacle as well as in a driven active fluid, for which activity is modeled with an Ornstein–Uhlenbeck process applied to each particle. The global dissipation is calculated over the entire system and is associated, in most cases, with a vanishingly low probability of observing trajectories with a negative dissipation. On the other hand, local dissipation is calculated over a small subregion of the system and exhibits a non-zero fraction of trajectories with a negative dissipation or negative entropy production. We connect the results across the local and global scales by showing that, while global dissipation follows the conventional fluctuation theorem, local dissipation in highly inhomogeneous and active matter systems satisfies a local version of the theorem. We further quantify the impact of localization on local entropy production through the correlations between the local region and the rest of the system. For the driven active system, we isolate the contributions arising from the deterministic drive and from the activity to the total local dissipation. While both the active (stochastic) and the driven (deterministic) contributions both satisfy a local fluctuation theorem, we observe that localization impacts differently these two quantities. Correlations between the local region and the surroundings are found to be of opposite signs, resulting in an asymmetry function with a slope of less than 1 for the active contribution. This differs from the behavior of the asymmetry function for the field-induced contribution and for all other driven systems studied in this work. The mapping of local dissipation also reveals that, for small local regions and over short trajectories, the probability distribution for dissipation and entropy production exhibits a negative tail, corresponding to trajectories where entropy is essentially consumed rather than produced. This finding is in line with experimental observations of entropy-consuming trajectories for colloidal particles in an optical trap over time scales on the order of seconds. These results are crucial to the operation of nanomachines and for the understanding of how protein motors operate. As thermodynamic engines become smaller and their time of operation becomes shorter, the probability for nanoengines to run thermodynamically in reverse will increase dramatically.^20,51^ Furthermore, recent advances in experimental methods, most notably in microrheology,^71,72^ are poised to allow researchers in the field to bridge the behavior of living systems with the theoretical analysis presented in this work. Optical and magnetic tweezers offer a unique way to study complex systems at the single-molecule level and have enabled experimental validation of the fluctuation theorem. For example, the fluctuation theorem has been demonstrated to hold for colloidal particles in an optical trap by analyzing their steady-state trajectories,^20,51^ in RNA hairpin systems,^12^ and for the F_1_-ATPase motor protein.^53,54^ It is anticipated that active microrheology and particle tracking will provide access to the spectrum of dissipated energy for a wide range of living and active systems.^73,74^ Future work will focus on applying this framework to unravel the onset of complex collective behaviors in active matter, most notably to the analysis of motility-induced phase separation^28,45^ and stimulus-driven pattern formation in bacteria.^24^

## Supplementary Material











## Notes

Mathematical derivations and simulation details (PDF)
Nonequilibrium response of the first system (Movie M1) (MP4)
Nonequilibrium response of the second system (Movie M2) (MP4)
Nonequilibrium response of the third system (Movie M3) (MP4)
Transparent Peer Review report available (PDF)
