# Supplementary material for: Mapping Local Dissipation and Entropy Production in Complex and Active Fluids
Source: J Phys Chem Lett. 2025 Oct 24;16(44):11405–13. doi: 10.1021/acs.jpclett.5c02469 (PMC12598859; doi:10.1021/acs.jpclett.5c02469)
Supplement: Supplementary file 1 [file jz5c02469_si_001.pdf]

SI: Mapping local dissipation and entropy production in complex and active fluids

Caroline Desgranges*,† and Jerome Delhommelle*,‡

†*Department of Physics and Applied Physics, University of Massachusetts, Lowell, MA 01854, USA*

‡*Department of Chemistry, University of Massachusetts, Lowell, MA 01854, USA*

E-mail: caroline_desgranges@uml.edu; jerome_delhommelle@uml.edu

Phone: 978-934-3721; 978-934-4367

1. Derivation of the expressions for dissipation and entropy production

1.1. Fluid driven in a channel of varying width

The equations of motion for the wall and fluid particles are given by

$$\begin{aligned} \dot{\mathbf{q}}_i &= \frac{\mathbf{p}_i}{m_i} \\ \dot{\mathbf{p}}_i &= \mathbf{F}_i + c_i F_e \mathbf{e}_x - S_i \left[\alpha \mathbf{p}_i + k\left(\mathbf{q}_i - \mathbf{q}_{0,i}\right)\right] \end{aligned} \tag{1}$$

in which $\mathbf{q}_i$, $\mathbf{p}_i$, m_i, and $c_i = (-1)^i$ denote the position, momentum, mass ($m_i = 1$ for all particles) and color charge of particle i ($c_i = 0$ for a wall particle), S_i a parameter equal to 1 for a wall particle and 0 otherwise, $\mathbf{F}_i$ the force exerted on particle i by the other particles,

F_e the strength of the color field exerted along the x axis of unit vector $\mathbf{e}_x$, k the spring constant that tethers the wall atom to its lattice position $\mathbf{q}_{0,i}$.

To calculate dissipation, we need to evaluate the rate of change of the Hamiltonian $\mathcal{H}$, which corresponds to the rate of change of the internal energy, and the phase space compression factor $\Lambda(\mathbf{\Gamma})$. The internal energy of the system is defined as[1]

$$\mathcal{H} = \sum_i \frac{\mathbf{p}_i^2}{2m_i} + \frac{1}{2}\sum_i \sum_{j \neq i} \phi(q_{ij}) + \sum_i \frac{k}{2} S_i \left(\mathbf{q}_i - \mathbf{q}_{0,i}\right)^2 \tag{2}$$

The rate of change in internal energy is given by

$$\dot{\mathcal{H}} = \sum_i \frac{\dot{\mathbf{p}}_i \cdot \mathbf{p}_i}{m_i} + \sum_i \dot{\mathbf{q}}_i \left[\nabla_i \sum_{j \neq i} \phi(q_{ij})\right] + \sum_i k S_i \dot{\mathbf{q}}_i \cdot (\mathbf{q}_i - \mathbf{q}_{0,i}) \tag{3}$$

Denoting by $\mathbf{F}_i = -\nabla_i \sum_{j \neq i} \phi(q_{ij})$ the force exerted on particle i by the other particles $(j \neq i)$, we obtain

$$\dot{\mathcal{H}} = \sum_i \frac{\dot{\mathbf{p}}_i \cdot \mathbf{p}_i}{m_i} - \sum_i \mathbf{F}_i \cdot \frac{\mathbf{p}_i}{m_i} + \sum_i k S_i \frac{\mathbf{p}_i}{m_i} \cdot (\mathbf{q}_i - \mathbf{q}_{0,i}) \tag{4}$$

Replacing $\dot{\mathbf{p}}_i$ by the second line of Eq. 1, we obtain

$$\begin{aligned} \dot{\mathcal{H}} &= \sum_i \frac{c_i F_e p_{x,i}}{m_i} - \sum_i S_i \frac{\alpha \mathbf{p}_i^2}{m_i} \\ &= F_e J_x - 2\alpha K_w \end{aligned} \tag{5}$$

in which $J_x = \sum_i c_i p_{x,i}/m_i$ denotes the instantaneous current along the $\mathbf{x}$ axis, K_w denotes the kinetic energy of the wall with $K_w = N_w/\beta$ according to the equipartition principle. We

now turn to the phase space compression factor $\Lambda(\mathbf{\Gamma})$ given by

$$\begin{aligned}\Lambda(\mathbf{\Gamma}) &= \frac{\partial}{\partial \mathbf{\Gamma}} \cdot \dot{\mathbf{\Gamma}} \\ &= \sum_i \frac{\partial \dot{\mathbf{q}}_i}{\partial \mathbf{q}_i} + \sum_i \frac{\partial \dot{\mathbf{p}}_i}{\partial \mathbf{p}_i} \\ &= -2\sum_i \alpha S_i \\ &= -2N_w\alpha\end{aligned} \tag{6}$$

We obtain the following expression for the instantaneous dissipation function $\Omega(\mathbf{\Gamma}(t))$

$$\begin{aligned}\Omega(\mathbf{\Gamma}(t)) &= \beta\frac{d\mathcal{H}(\mathbf{\Gamma}(t))}{dt} - \Lambda(\mathbf{\Gamma}(t)) \\ &= \beta J_x F_e\end{aligned} \tag{7}$$

since $\beta K_w = N_w$.

The local dissipation Ω_L in a region $\mathcal{L}$ characterized by a length L is given by

$$\Omega_L = \beta J_{x,L} F_e \tag{8}$$

in which $J_{x,L} = \sum_{i\in\mathcal{L}} c_i p_{x,i}/m_i$

The dissipation Ω_t measured over a trajectory of duration t is obtained by integrating Eq. 7 with respect to time as follows

$$\Omega_t = \int_0^t \Omega(\mathbf{\Gamma}(t))dt \tag{9}$$

and averaged over a large set of trajectories. Similarly, the local dissipation $\Omega_{L,t}$ is obtained by performing the following integration

$$\Omega_{L,t} = \int_0^t \Omega_L(\mathbf{\Gamma}(t))dt \tag{10}$$

1.2. Fluid driven past a fixed obstacle

The equations of motion for the fixed obstacle are given by

$$\begin{aligned}\dot{\mathbf{q}}_i &= \frac{\mathbf{p}_i}{m_i} \\ \dot{\mathbf{p}}_i &= \mathbf{F}_i + c_i F_e \mathbf{e}_x - \alpha \mathbf{p}_i\end{aligned} \tag{11}$$

in which the force $\mathbf{F}_i$ exerted on the particle i is the sum of contributions from interparticle and obstacle-particle interactions. α is a Gaussian ergostat multiplier that keeps the internal energy constant. The rate of change in internal energy is given by

$$\begin{aligned}\dot{\mathcal{H}} &= \sum_i \frac{\dot{\mathbf{p}}_i \cdot \mathbf{p}_i}{m_i} + \sum_i \dot{\mathbf{q}}_i \left[\nabla_i \sum_{j \neq i} \phi(q_{ij}) \right] \\ &= J_x F_e - \alpha \sum_i \frac{\mathbf{p}_i^2}{m_i} \\ &= J_x F_e - \frac{2N\alpha}{\beta} \\ &= 0\end{aligned} \tag{12}$$

in which, in line with the previous example, we have plugged in the second line of Eq, 11 for $\dot{\mathbf{p}}_i$, used the equipartition principle for $\beta \sum_i^N p_i^2 = 2N$, and used the fact that the internal energy is kept constant. The first term corresponds to the rate at which work is performed on the system ($\dot{W} = J_x F_e$) and the second term to the rate at which heat is absorbed by the system.

The phase space compression factor is given by

$$\Lambda(\mathbf{\Gamma}) = \frac{\partial}{\partial \mathbf{\Gamma}} \cdot \dot{\mathbf{\Gamma}} = -2N\alpha \tag{13}$$

We then obtain the instantaneous dissipation function $\Omega(\mathbf{\Gamma}(t))$

$$\Omega(\mathbf{\Gamma}(t)) = -\Lambda(\mathbf{\Gamma}(t)) = 2N\alpha = \beta J_x F_e \tag{14}$$

We recover a similar expression for the dissipation to that found for the previous system, except that the temperature is no longer constant and needs to be numerically integrated. Furthermore, since the system is adiabatic, the rate at which work is performed on the system is equal to the rate of the heat flow. We therefore obtain

$$\Omega(\mathbf{\Gamma}(t)) = \beta(\mathbf{\Gamma}(t))\dot{Q} = \dot{\Sigma}(\mathbf{\Gamma}(t)) \tag{15}$$

where $\dot{\Sigma}(\mathbf{\Gamma}(t))$ denotes the rate of entropy production.

The entropy production over a trajectory of duration t is then obtained as

$$\Sigma_t = \int_0^t \beta(\mathbf{\Gamma}(t)) J_x F_e dt \tag{16}$$

and local entropy production follows as

$$\Sigma_{L,t} = \int_0^t \beta(\mathbf{\Gamma}(t)) J_{x,L} F_e dt \tag{17}$$

1.3. Driven active fluid

We also test our approach on an active fluid driven by a color field. As in the AOUP model,[2] we model the activity of a particle by adding an Ornstein-Uhlenbeck process along the x axis to the equation of motion for the momentum. These equations are thus given by

$$\begin{aligned} \dot{\mathbf{q}}_i &= \frac{\mathbf{p}_i}{m_i} \\ \dot{\mathbf{p}}_i &= \mathbf{F}_i + c_i F_e \mathbf{e}_x + \dot{\xi}_i \mathbf{e}_x - \alpha \mathbf{p}_i \\ \dot{\xi}_i &= -\frac{\xi_i}{\tau} + \eta_i \end{aligned} \tag{18}$$

In this case, α is chosen to act as a thermostat multiplier that keeps temperature constant. Applying the same analysis as in the previous sections, we find that dissipation is given by

$$\Omega(\mathbf{\Gamma}(t)) = \beta F_e J_x + \sum_i \xi_i p_{x,i} \tag{19}$$

$\Omega(\mathbf{\Gamma}(t))$ is thus the sum of a deterministic term, resulting from the action of the external field on the particles, and of an active stochastic term, resulting from the action of the OUP processes applied to each of the particles. The dissipation measured over a trajectory of duration t is then given by

$$\begin{aligned}\Omega_t =& \int_0^t \beta F_e J_x dt + \int_0^t \sum_i \xi_i p_{x,i} dt \\ =& \Omega_{F_e,t} + \Omega_{\xi,t}\end{aligned} \tag{20}$$

in which the first term denotes the deterministic contribution $\Omega_{F_e,t}$ and the second term the active stochastic contribution $\Omega_{\xi,t}$.

This gives the following expression for the local dissipation $\Omega_{L,t}$

$$\begin{aligned}\Omega_{L,t} =& \int_0^t \beta F_e J_{x,L} dt + \int_0^t \sum_{i\in\mathcal{L}} \xi_i p_{x,i} dt \\ =& \Omega_{L,F_e,t} + \Omega_{L,\xi,t}\end{aligned} \tag{21}$$

2. Simulation parameters and details

All particles have the same mass m. Interactions between wall and/or fluid particles are modeled by a functional form based on the WCA potential[3] and are given by

$$\phi(r_{ij}) = 4\epsilon\left[\left(\frac{\sigma}{r_{ij}}\right)^{12} - \left(\frac{\sigma}{r_{ij}}\right)^{6}\right] + \epsilon \tag{22}$$

in which r_{ij} is the distance between two particles i and j, and σ and ϵ denote the exclusion diameter and the energy parameter, respectively, for the WCA potential. Throughout the manuscript, the simulation parameters and results are given in the conventional system of reduced units[4] with σ as the unit of time, ϵ as the unit of energy, and m as the unit of mass. We use a spherical cutoff r_c for the calculations of the interactions $r_c = 2^{1/6}\sigma$, since the WCA potential and its first derivative both vanish at $r = r_c$. In the second system, particles are driven past a disk-shaped obstacle. In this case, the interaction between a fluid particle and the obstacle is given by a modified WCA potential[5,6]

$$\phi(r_{ij}) = 4\epsilon\left[\left(\frac{D}{r_{ij} - \nu_{ij}}\right)^{12} - \left(\frac{D}{r_{ij} - \nu_{ij}}\right)^{6}\right] + \epsilon \tag{23}$$

in which D denotes the exclusion diameter for the interaction. Interactions are calculated for distances below the cutoff distance, i.e., for $r_{ij} < (\nu_{ij} + 2^{1/6}D)$, and equal to 0 otherwise. In Eq. 23, ν_{ij} is equal to $R_i + R_j - D$ in which R_i and R_j are the radii of particles i and j, respectively. The radius of a fluid particle is set to 0.5σ, while the radius for the obstacle is set to 5σ.

The dissipation function and entropy production are integrated over 10^5 nonequilibrium molecular dynamics trajectories that start from an equilibrium configuration of the system, *i.e.*, with the external field and activity switched off. In line with previous work,[1,7] the equations of motion are propagated with a fourth-order Runge-Kutta integrator with a timestep of 5×10^{-4} time units. We use the following simulation parameters. For the first system (particles driven in a channel of varying width), we consider a rectangular simulation cell with $L_x = 50.6$ and $L_y = 10.8$, $N_{wall} = 132$, placed on a 2D face-centered cubic lattice, $N_{fluid} = 158$, a spring constant for the wall potential $k = 100$, an external field set to $F_e = 0.3$, a wall temperature of 1, and a trajectory duration of 5 time units. For the second system (particles driven past a disk-shaped obstacle), we now have a square simulation cell of edge L with a fluid density of $N_{fluid}/L^2 = 0.4$ and a number of fluid particles set to

$N_{fluid} = 200$, an external field set to $F_e = 0.2$, a temperature of 1, and a trajectory duration of 5 time units. Finally, for the third system (driven active particles), we also consider a square simulation cell and a fluid density of $N_{fluid}/L^2 = 0.4$, with a number of fluid particles set to $N_{fluid} = 200$, an external field set to $F_e = 0.05$, a temperature of 1, and a trajectory duration of 3 time units. For the OUP process applied to each particle, we set the standard deviation to $\sigma = 2F_e$ and the relaxation time to $\tau = 0.1$. Finally, we use the conventional periodic boundary conditions are also applied to all systems.[4]

References

(1) Evans, D.; Morriss, G. *Nonequilibrium Statistical Mechanics of Liquids*; Cambridge University Press, Cambridge, 2008.

(2) Nguyen, G. P.; Wittmann, R.; Löwen, H. Active Ornstein–Uhlenbeck model for self-propelled particles with inertia. *J. Phys. Condens. Matt.* **2021**, *34*, 035101.

(3) Weeks, J. D.; Chandler, D.; Andersen, H. C. Role of repulsive forces in determining the equilibrium structure of simple liquids. *J. Chem. Phys.* **1971**, *54*, 5237–5247.

(4) Allen, M. P.; Tildesley, D. J. *Computer Simulation of Liquids*; Clarendon, Oxford, 1987.

(5) Delhommelle, J.; Petravic, J. Shear thickening in a model colloidal suspension. *J. Chem. Phys.* **2005**, *123*, 074707.

(6) Desgranges, C.; Delhommelle, J. Entropy production in model colloidal suspensions under shear via the fluctuation theorem. *J. Chem. Phys.* **2020**, *153*.

(7) Desgranges, C.; Delhommelle, J. Molecular simulation of transport in nanopores: Application of the transient-time correlation function formalism. *Phys. Rev. E* **2008**, *77*, 027701.
