# Supplementary material for: Mapping Local Dissipation and Entropy Production in Complex and Active Fluids
Source: J Phys Chem Lett. 2025 Oct 24;16(44):11405–13. doi: 10.1021/acs.jpclett.5c02469 (PMC12598859; doi:10.1021/acs.jpclett.5c02469)
Supplement: Supplementary file 5 [file jz5c02469_si_005.pdf]

jz-2025-02469d.R1

Name: Peer Review Information for "Mapping local dissipation and entropy production in complex and active fluids"

First Round of Reviewer Comments

Reviewer: 1

Comments to the Author

The article by Desgranges and Delhommelle addresses a topic of extraordinary importance for the physical chemistry community: the possibility of spatially mapping local dissipation and entropy production in complex systems and active fluids. The work is fully in line with the growing interest in non-equilibrium thermodynamics, a field which, despite decades of theoretical progress, still presents numerous challenges, especially with regard to flow and heat transport phenomena. The authors propose a robust protocol, rooted in the formalism of the local dissipation theorem, and demonstrate its effectiveness through simulations on confined fluids and active systems. The results are solid, well presented and supported by a clear connection with the fluctuation theorem.

In my opinion, the manuscript deserves publication after some minor revisions that could further strengthen its impact. In particular, I would identify three points on which the authors could intervene to make the contribution even more relevant to the scientific community:

1. The article focuses heavily on simulation approaches and the local formulation of the dissipation theorem, but lacks an explicit comparison with classical concepts of non-equilibrium thermodynamics (Prigogine, De Groot-Mazur, flow-force relations, etc.). It is not clarified how the proposed local formulation fits into the broader framework of the theory of irreversible processes. This is crucial in order to align the results with established tradition and provide experimentalists with a bridge between numerical simulation and macroscopic observables. Including even a single paragraph of discussion relating the

results to classical formalism would give the article greater relevance for the physical chemistry community and emphasise the urgency of progress in this area.

2. The text shows distributions of local entropy dissipation/production with negative tails, but the discussion is limited to confirming that this is consistent with the local fluctuation theorem. What this means physically for the system is not explored in depth: are these transient events? Statistical fluctuations? Or is there a possible interpretation related to local work extraction? The possibility of having regions with negative entropy production is an aspect that attracts a lot of attention in the community: clarifying the physical meaning of these events is essential. A more extensive discussion would give depth to the results, opening up perspectives on applications in nanomachines, biology and out-of-equilibrium systems, as well as highlighting the connection between theory and possible experimental verifications.

3. The article remains confined to the simulation level, with an elegant protocol but no clear indications on how the methodology could be experimentally verified or exploited in applications (e.g., in active biological fluids, nanofluidics, or soft matter systems observable with optical or spectroscopic techniques). The physical chemistry community is in dire need of tools that connect simulations and real measurements: local heat flows, spectral imaging of dissipation, microrheology experiments, etc.

Adding even just a section on prospects, with possible experimental techniques to validate the method, would greatly increase the impact of the work and highlight its urgency in terms of advancing research on non-equilibrium thermodynamics.

Reviewer: 2

Comments to the Author

In this manuscript by Desgranges and Delhommelle, the authors apply the framework of Michel and Searles to map local entropy production in particle-based simulations. This is an important and difficult problem: how to define and measure local entropy production in nonequilibrium systems. The theoretical framework is rigorous, connects naturally to fluctuation theorem literature, and addresses the limitation that global entropy production

averages out local effects. The results on two model systems are interesting and the approach is reasonable. With further development, this could be of value to the active matter community.

That said, I found several issues that limit the impact of the work in its present form:

- Several key claims are stated in general terms (‘robust framework’, ‘unique response’) but not substantiated with either data or a precise definition. I recommend the authors sharpen these statements and ensure that their abstract and conclusion reflect the findings.
    - The claim of a ‘unique nonequilibrium response’ is not clearly defined or demonstrated. The authors should specify what response they mean and show explicitly how it appears in the results.
    - "The results establish that our approach provides reliable and physically meaningful measures for local dissipation, even when the system is highly inhomogeneous." The claim that the measures are ‘physically meaningful’ could be substantiated further. Without comparison to experiment or other validated models, this remains more of an assumption than a demonstrated fact.
    - “The results obtained in this work show that even if the driven active system is still in the linear regime, it no longer acts as if it were in equilibrium.” The discussion of this point is terse, and should be expanded—why would one expect equilibrium-like behavior?
- There are now several approaches to quantifying dissipation in active matter. The authors should better position their approach against these efforts, making the advantages and limitations explicit:
    - A partial list:
        - https://www.nature.com/articles/s41467-019-09631-x
        - https://doi.org/10.1073/pnas.2024300118
        - https://www.nature.com/articles/s41467-019-11051-w
        - https://doi.org/10.1126/science.aac8167
        - https://www.pnas.org/doi/10.1073/pnas.2318106121
        - https://doi.org/10.1103/PhysRevResearch.7.L012078

- https://www.pnas.org/doi/full/10.1073/pnas.2017042117

- https://www.pnas.org/doi/10.1073/pnas.2207662120

- The authors use deterministic, time-reversible thermostats. However, as they say on p. 4 existing models for active matter "generally include both stochastic and deterministic terms in their equations of motion". Langevin dynamics with a fixed friction coefficient for the amplitude of the thermal noise are common. Random forces in the dynamics wouldn't prevent the analysis of local contributions to dissipation or local fluctuation theorems in stochastic thermodynamics.

- Using Gaussian thermostats makes dissipation accounting rigorous and fluctuation theorem tests clean, but does this come at the cost of physical meaning without random bath noise or a canonical distribution at equilibrium?

- The thermostats here are necessary to remove excess heat generated by external driving, keeping the system in a steady state. But, could the authors articulate the reasons for using a *deterministic* thermostat?

- Aren't the dissipation function and, so, the entropy production and fluctuation theorem, thermostat dependent? I expect results here to change to some extent, even for another purely deterministic thermostats (e.g., Nosé–Hoover versus Gaussian isoenergetic).

- Since the central claim is spatially resolved dissipation, the absence of actual dissipation profiles (e.g., as a function of horizontal position) weakens the manuscript. Including such visualizations would make the contribution much more compelling.

- Similarly, the bin definition is important to the analysis; the authors should explain how bins were chosen and justify the bin width of 4.6.

- The examples are highly idealized (fluids of repulsive disks). While they provide conceptual clarity, it is not obvious how these correspond to experimental active matter systems. If the paper's claim is to address ‘complex and active fluids,’ the authors should articulate which experimental phenomena these models are meant to capture and why the present framework provides unique insight into them.

- The authors also conclude with "Future work will focus on applying this framework to unravel the onset of complex collective behaviors in active matter." Since the title emphasizes active matter, it would be helpful if the authors made clearer which aspects of experimental active matter their model systems are intended to represent.

Overall, the framework is rigorous and the results are interesting, but the manuscript in its current form does not yet rise to the level of clarity of a Letter. I encourage the authors either to strengthen the main text (by clarifying claims, explicitly mapping spatial dissipation, and contextualizing their results relative to experimental active matter), or to consider expanding the manuscript into a regular article where more methodological and numerical details can be developed.

Author's Response to Peer Review Comments:

Response to the reviewers and changes made to the manuscript

Mapping local dissipation and entropy production in complex and active fluids

Caroline Desgranges[1] and Jerome Delhommelle[2]

[1]Department of Physics & Applied Physics, University of Massachusetts, Lowell, MA 01854, USA [2]Department of Chemistry, University of Massachusetts, Lowell, MA 01854, USA

Reviewer #1

The article by Desgranges and Delhommelle addresses a topic of extraordinary importance for the physical chemistry community: the possibility of spatially mapping local dissipation and entropy production in complex systems and active fluids. The work is fully in line with the growing interest in non-equilibrium thermodynamics, a field which, despite decades of theoretical progress, still presents numerous challenges, especially with regard to flow and heat transport phenomena. The authors propose a robust protocol, rooted in the formalism of the local dissipation theorem, and demonstrate its effectiveness through simulations on confined fluids and active systems. The results are solid, well presented and supported by a clear connection with the fluctuation theorem.

In my opinion, the manuscript deserves publication after some minor revisions that could further strengthen its impact. In particular, I would identify three points on which the authors could intervene to make the contribution even more relevant to the scientific community:.

Author reply: We thank the reviewer for their positive comments.

---

1. The article focuses heavily on simulation approaches and the local formulation of the dissipation theorem,but lacks an explicit comparison with classical concepts of non-equilibrium thermodynamics (Prigogine, De Groot-Mazur, flow-force relations, etc.). It is

not clarified how the proposed local formulation fits into the broader framework of the theory of irreversible processes. This is crucial in order to align the results with established tradition and provide experimentalists with a bridge between numerical simulation and macroscopic observables. Including even a single paragraph of discussion relating the results to classical formalism would give the article greater relevance for the physical chemistry community and emphasise the urgency of progress in this area.

Author reply: We thank the reviewer for this excellent point and have added a paragraph on how entropy pro-

---

duction, which quantifies irreversibility, also serves as a bridge between microscopic dissipation phenomena and macroscopic observables. We provide below in blue font the corresponding paragraph.

The extension of the concepts of equilibrium thermodynamics, which include, among others, the definition of entropy through the second law of thermodynamics, to nonequilibrium systems has drawn considerable interest in recent decades. The assumption of local equilibrium allows for the determination of explicit expressions for entropy production as products of generalized forces (affinities, chemical potential gradients, or temperature gradients) by the rates at which irreversible processes (chemical reactions, diffusion, or heat flows) occur [1]. Although this approach provides a link between entropy production and measurable fluxes and forces, it remains limited to systems that are near equilibrium and where linear relations between flows and forces hold. For far-from-equilibrium systems, recent progress in nonequilibrium statistical mechanics has shown how the theory of dynamical systems leads to a quantitative determination of entropy production [2]. This theory reveals that, for systems that satisfy the chaotic hypothesis [3], entropy production is directly related to phase space contraction and thus to the rate at which dissipative work is performed on the system [4]. The fluctuation theorem [3, 5, 6] and its generalized form known as dissipation theorem [7, 8], provide an expression for the probability distribution of the dissipation, or generalized entropy production, and have been experimentally verified by following the trajectory of a colloidal particle captured in an optical trap [9, 10].

2. The text shows distributions of local entropy dissipation/production with negative tails, but the discussionis limited to confirming that this is consistent with the local fluctuation theorem. What this means physically for the system is not explored in depth: are these transient events? Statistical fluctuations? Or is there a possible interpretation related to local work extraction? The possibility of having regions with negative entropy production is an aspect that attracts a lot of attention in the community: clarifying the physical meaning of these events is essential. A more extensive discussion would give depth to the results, opening up perspectives on applications in nanomachines, biology and out-of-equilibrium systems, as well as highlighting the connection between theory and possible experimental verifications.

Author reply: We thank the reviewer for bringing up this point and have added a paragraph to discuss

the meaning of negative tails in the probability distribution, their impact on nanomachines and biological systems, and the connection with experimental findings. We provide below in blue font a copy of the added paragraph.

The mapping of local dissipation reveals that, for small local regions and over short trajectories, the probability distribution for dissipation and entropy production exhibits a negative tail, corresponding to trajectories where entropy is essentially consumed rather than produced. This finding is in line with experimental observations of entropy-consuming trajectories for colloidal particles in an optical trap over time scales on the order of seconds. These results are crucial to the operation of nanomachines and for the understanding of how protein motors operate. As thermodynamic engines become smaller and their time of operation becomes shorter, the probability for nanoengines to run thermodynamically in reverse will increase dramatically [9, 10].

3. The article remains confined to the simulation level, with an elegant protocol but no clear indications onhow the methodology could be experimentally verified or exploited in applications (e.g., in active biological fluids, nanofluidics, or soft matter systems observable with optical or spectroscopic techniques). The physical chemistry community is in dire need of tools that connect simulations and real measurements: local heat flows, spectral imaging of dissipation, microrheology experiments, etc. Adding even just a section on prospects, with possible experimental techniques to validate the method, would greatly increase the impact of the work and highlight its urgency in terms of advancing research on non-equilibrium thermodynamics.

Author reply: We thank the reviewer for this excellent suggestion. We have added a paragraph to the conclusion to discuss how recent experimental developments, such as, *e.g.*, in microrheology, can bridge with the theoretical analysis discussed in this work. We provide in blue the added paragraph below.

Furthermore, recent advances in experimental methods, most notably in microrheology [11, 12], are poised to allow researchers in the field to bridge the behavior of living systems with the theoretical analysis presented in this work. Optical and magnetic tweezers offer a unique way to study complex systems at the single-molecule level and have enabled experimental validation of the fluctuation theorem. For example, the fluctuation theorem has been demonstrated to hold for colloidal particles in an optical trap by analyzing their steady-state trajectories [9, 10], in RNA hairpin systems [13], and for the F_1-ATPase motor protein [14, 15]. It is anticipated that active microrheology and particle-tracking will provide access to the spectrum of dissipated energy for a wide range of living and active systems [16, 17].

Reviewer #2

In this manuscript by Desgranges and Delhommelle, the authors apply the framework of Michel and Searles to map local entropy production in particle-based simulations. This is an important and difficult problem: how to define and measure local entropy production in nonequilibrium systems. The theoretical framework is rigorous, connects naturally to fluctuation theorem literature, and addresses the limitation that global entropy production averages out local effects. The results on two model systems are interesting and the approach is reasonable. With further development, this could be of value to the active matter community.

Author reply: We thank the reviewer for their positive comments.

That said, I found several issues that limit the impact of the work in its present form:
- Several key claims are stated in general terms ('robust framework', 'unique response') but not substantiated with either data or a precise definition. I recommend the authors sharpen these statements and ensure that their abstract and conclusion reflect the findings.

Author reply: We thank the reviewer for drawing our attention to this point. We mean by 'robust' that the

---

proposed framework is applicable to fluid flows in complex environments (channels of varying width and flows past obstacles) and to flows of active particles. By 'unique response', we mean that the active contribution to local dissipation exhibits a different behavior, with a slope below 1 in the plot for the local fluctuation theorem in Fig. 5, when compared to the field-driven contribution and to the total local dissipation. We have modified the abstract and the conclusion accordingly and provide in blue the corresponding definitions and amended statements.

For the abstract: While global entropy production provides a measure of irreversibility, its partitioning into contributions from local regions is key to understanding the mechanisms underlying time-reversal symmetry breaking in complex systems and active matter. Here, by analyzing local heat flows and fluxes, we propose a framework that enables the mapping of local dissipation and entropy production in a nonequilibrium system. We test this approach in simulations of fluids driven through complex environments and of a driven active fluid. We connect the results across the local and global scales by showing that local dissipation and entropy production satisfy a local version of the usual (global) fluctuation theorem, which accounts for the correlations between the local region and its surroundings. Interestingly, while the local fluctuation theorem holds for the driven active fluid, our analysis reveals the presence of correlations of opposite signs for the active and field-induced contributions to local dissipation.

In the conclusion: We assess the reliability of our approach by mapping the local dissipation and entropy production in fluids driven through complex environments, *i.e.*, through nanochannels of varying width or past a fixed obstacle, as well as in a driven active fluid, for which activity is modeled with an OrnsteinUhlenbeck process applied to each particle.

Also, in the conclusion: For the driven active system, we isolate the contributions arising from the deterministic drive and from the activity to the total local dissipation. While the active (stochastic) and the driven (deterministic) contributions both satisfy a local fluctuation theorem, we observe that localization impacts differently these two quantities. Correlations between the local region and the surroundings are found to be of opposite signs, resulting in an asymmetry function with a slope of less than 1 for the active contribution. This differs from the behavior of the asymmetry function for the field-induced contribution and for all other driven systems studied in this work.

- *The claim of a 'unique nonequilibrium response' is not clearly defined or demonstrated. The authors should specify what response they mean and show explicitly how it appears in the results.*

Author reply: We have clarified this statement in the abstract, main text, and conclusion. In the main text,

we have amended the discussion of the results shown in Fig. 5 to explain the difference in behavior observed for the active (stochastic) contribution to local dissipation when compared to the field-driven (deterministic) contribution. We provide in blue the corresponding explanation.

For the discussion of Fig. 5, Very interestingly, the total local dissipation, as well as the deterministic and active stochastic contributions, all exhibit a linear asymmetry function. While both the active (stochastic) and the driven (deterministic) terms thus satisfy a local fluctuation theorem, we observe that localization impacts differently the two contributions. For the active part, we find that the slope for the asymmetry function is below 1 (0.64 ± 0.03), unlike what is observed for the deterministic contribution and for the total local dissipation in the case of this system and for the local dissipation obtained for the first two driven systems studied here. This means that, in the case of the active contribution, the sign of the local factor $\kappa(L)$ becomes negative, which indicates a negative correlation between the local region and its surroundings in marked contrast with the observed behavior for the deterministic contribution and the other deterministic systems.

We have also modified the abstract to better explain the difference in response for the active and field-driven contributions: Interestingly, while the local fluctuation theorem holds for the driven active fluid, our analysis reveals the presence of correlations of opposite signs for the active and field-induced contributions to local dissipation.

In the conclusion, the modified statement now reads: For the driven active system, we isolate the contributions arising from the deterministic drive and from the activity to the total local dissipation. While both the active (stochastic) and the driven (deterministic)

contributions both satisfy a local fluctuation theorem, we observe that localization impacts differently these two quantities. Correlations between the local region and the surroundings are found to be of opposite signs, resulting in an asymmetry function with a slope of less than 1 for the active contribution. This differs from the behavior of the asymmetry function for the field-induced contribution and for all other driven systems studied in this work.

- *"The results establish that our approach provides reliable and physically meaningful measures for local dissipation, even when the system is highly inhomogeneous." The claim that the measures are 'physically meaningful' could be substantiated further. Without comparison to experiment or other validated models, this remains more of an assumption than a demonstrated fact.*

Author reply: We thank the reviewer for the opportunity to clarify this point. The goal of this work is to

---

propose a spatial partition of the global dissipation into local contributions. Then, to prove that these local contributions provide a physically meaningful measure of local dissipation, we verify that these local contributions satisfy the well-established fluctuation theorem. The validity of the fluctuation theorem was established using dynamical systems theory and the Sinai-Ruelle-Bowen measures and verified experimentally for several colloidal and living systems. We provide in blue the corresponding discussion.

The following statements were added to the introduction.

First, we have included the point raised by the reviewer as a question: How can we assess that a given spatial partition of the global dissipation into local contributions provides a physically meaningful measure of local dissipation?

We have then included the following statement in response to this question:

To establish that the proposed spatial partition of the global dissipation into local contributions provides a physically meaningful measure of local dissipation, we verify that these local contributions satisfy the dissipation and fluctuation theorems [3, 5, 6, 18], which was established using dynamical systems theory and the Sinai-Ruelle-Bowen measures [2, 3, 5] and validated experimentally for several colloidal [9, 10, 15] and living systems [13, 14].

- *"The results obtained in this work show that even if the driven active system is still in the linear regime, it no longer acts as if it were in equilibrium." The discussion of this point is terse, and should be expanded-why would one expect equilibrium-like behavior?*

Author reply: We agree with the reviewer that the statement was unclear. We have revised this statement

---

and expand statement the discussion of Fig. 5.

Very interestingly, the total local dissipation, as well as the deterministic and active stochastic contributions, all exhibit a linear asymmetry function. While both the active (stochastic) and the driven (deterministic) terms thus satisfy a local fluctuation theorem, we observe that localization impacts differently the two contributions. For the active part, we find that the slope for the asymmetry function is below 1 (0.64 ± 0.03), unlike what is observed for the deterministic contribution and for the total local dissipation in the case of this system and for the local dissipation obtained for the first two driven systems studied here. This means that, in the case of the active contribution, the sign of the local factor $\kappa(L)$ becomes negative, which indicates a negative correlation between the local region and its surroundings in marked contrast with the observed behavior for the deterministic contribution and the other deterministic systems.

- *There are now several approaches to quantifying dissipation in active matter. The authors should better position their approach against these efforts, making the advantages and limitations explicit. A partial list:*

https://www.nature.com/articles/s41467-019-09631-x

https://www.pnas.org/doi/abs/10.1073/pnas.2024300118 https://www.nature.com/articles/s41467-019-11051-w

https://www.science.org/doi/full/10.1126/science.aac8167?casa_token=9rQT3nQhvfYAAAA 3AVcn8GdnOk2s8B75k4hNsJU3yiqyg7w0CPlYDdjoBUBrjaO8EcGBQUDTgWzjv49XeVQJSp3b0cxFV3g

https://www.pnas.org/doi/abs/10.1073/pnas.2318106121

https://journals.aps.org/prresearch/abstract/10.1103/PhysRevResearch.7.L012078

https://www.pnas.org/doi/abs/10.1073/pnas.2017042117 https://www.pnas.org/doi/abs/10.1073/pnas.2207662120

Author reply: We thank the reviewer for drawing our attention to these insightful papers. We have included

---

a discussion of these papers in the introduction and provide in blue the corresponding statements

The approach introduced in this work builds upon recent efforts to quantify dissipation in complex and active fluids by offering a thermodynamically consistent and spatially resolved framework that links microscopic simulation data to local entropy production measures. The determination of local entropy production in living systems has recently been the focus of intense research. Following the pioneering analysis of broken detailed balance in an active biological system using video microscopy and statistical physics tools [19], several novel approaches were developed to evaluate entropy production rates from experimental data. These methods relied on using fluctuating currents and the thermodynamic uncertainty relation [20], leveraging estimators based on time asymmetry in waiting times in the absence of currents [21], inferring dissipation rates from experimental data using the Fisher information metric [22], or developing a mathematical framework to construct estimators that closely approximate the true entropy production

rate [23]. In recent years, machine learning models have also been leveraged to compute probability flows and entropy production using generative modeling [24] or to determine the mutual information between subsystems using a convolutional neural network [25]. These approaches complement experimental approaches that assessed the energetic efficiency of living systems by comparing the rate of energy consumption, measured using calorimetry, with the rate of energy dissipation through emergent flows, estimated from microscopy [26].

- *The authors use deterministic, time-reversible thermostats. However, as they say on p. 4 existing models for active matter "generally include both stochastic and deterministic terms in their equations of motion". Langevin dynamics with a fixed friction coefficient for the amplitude of the thermal noise are common. Random forces in the dynamics wouldn't prevent the analysis of local contributions to dissipation or local fluctuation theorems in stochastic thermodynamics.*

Author reply: We agree with the reviewer the analysis proposed here is also applicable to stochastic systems.

We have included two additional references in which the application of the fluctuation theorem to dissipative systems with both deterministic and stochastic components was discussed. We also highlight p. 4 that the third system studied in our work contains both deterministic and stochastic terms in the equations of motion with the addition of an Active Orstein-Uhlenbeck term.

The following statement was also added: We add that the fluctuation theorem is applicable to systems subjected to both stochastic and deterministic forces [4, 27] and consider in our analysis examples of deterministic systems for the first two systems studied in this work, as well as a system with both deterministic and stochastic forces in the third system considered in this work.

- *Using Gaussian thermostats makes dissipation accounting rigorous and fluctuation theorem tests clean, but does this come at the cost of physical meaning without random bath noise or a canonical distribution at equilibrium?*

Author reply: This is a very interesting point raised by the reviewer. We clarify that we examine two different

---

scenarios for heat removal in our work. While we apply a Gaussian thermostat to the fluid for the second and third systems, the first system uses a realistic model for heat removal, in which heat is removed through the boundaries. In this case, the Gaussian thermostat only applies to the atoms of the nanochannel walls. This means that the thermostat does not act on the fluid atoms and thus on the measured local dissipation. In this setup, the wall atoms play the role of a heat bath and the fluid is thermalized through the wall-fluid interactions. Very importantly, regardless of the scenario chosen for heat removal, the local dissipation we obtain satisfies a fluctuation theorem. To answer the last part of the reviewer's question, we add that the use of a Gaussian thermostat enables to recover a

canonical distribution at equilibrium for the configurational degrees of freedom [28]. We further discuss the impact of thermostats in response to another point below.

We have included the following statement after the discussion of the results obtained for the second system: Very interestingly, the results for the local entropy production, together with those obtained for the first system on local dissipation, reveal that the spatial partition proposed here holds for two very different mechanisms to account for heat dissipation. While we applied a Gaussian ergostat to the fluid for the second system, we used for the first system a realistic model for heat removal, since heat was removed through the boundaries. In the first system, the Gaussian thermostat only applied to the atoms of the nanochannel walls. This means that the thermostat did not act on the fluid atoms and thus on the measured local dissipation. Thus, the wall atoms played the role of a heat bath and the fluid was thermalized through the wall-fluid interactions. Regardless of the scenario chosen for heat removal, the spatial partition of the global dissipation and entropy production so obtained satisfies a fluctuation theorem in both cases.

- *The thermostats here are necessary to remove excess heat generated by external driving, keeping the system in a steady state. But, could the authors articulate the reasons for using a *deterministic* thermostat?*

Author reply: We thank the reviewer for this question. We clarify the reasons for using a deterministic

---

thermostat and provide a reference to Ruelle's analysis [2]. The deterministic thermostat we use in this study satisfies Gauss's principle of least constraint, which minimizes phase space compression and preserves time reversibility [29]. From a mathematical standpoint [2], using a Gaussian thermostat leads to a simple phase space dynamics, with the time evolution of the system taking place on a compact manifold, that can be analyzed with a Sinai-Ruelle-Bowen measure using the theory of smooth dynamical systems. Using a Gaussian thermostat thus provides a clear interpretation of dissipation and entropy production arising from external driving forces, and a rigorous and efficient framework for studying nonequilibrium steady states and their thermodynamic properties via phase space contraction.

We provide in blue the corresponding statement: This mechanism satisfies Gauss's principle of least constraint, which minimizes phase space compression and preserves time reversibility [29]. From a mathematical standpoint [2], this leads to a simple phase space dynamics, with the time evolution of the system taking place on a compact manifold, that can be analyzed with a Sinai-Ruelle-Bowen measure using the theory of smooth dynamical systems. Using a Gaussian thermostat thus provides a clear interpretation of dissipation and entropy production arising from external driving forces, and a rigorous and efficient framework for studying nonequilibrium steady states and their thermodynamic properties via phase space contraction.

Aren't the dissipation function and, so, the entropy production and fluctuation theorem, thermostat dependent? I expect results here to change to some extent, even for another purely deterministic thermostats (e.g., Nosé-Hoover versus Gaussian isoenergetic).

Author reply: We thank the reviewer for their comment. Although a given trajectory will show some sen-

---

sitivity to the thermostat details, the results for local entropy production and dissipation are independent from the thermostat details since they are averaged over many trajectories (typically 10^5 in the examples shown in the paper). We have included references to previous work by others who verified that the type of deterministic thermostat (Gaussian, Nosé-Hoover, and other examples) does not impact simulation results for global dissipation [30].

We have added the following statement: More generally, we add that the choice of a specific type of deterministic thermostat does not impact simulation results obtained for the dissipation and fluctuation theorem. In previous work, Williams *et al.* [30] study an infinite class of fictitious time-reversible deterministic thermostats and show that the fluctuation theorem is independent of the precise mathematical details of the thermostatting mechanism.

- *Since the central claim is spatially resolved dissipation, the absence of actual dissipation profiles (e.g., as a function of horizontal position) weakens the manuscript. Including such visualizations would make the contribution much more compelling.*

Author reply: We thank the reviewer for this excellent suggestion. We have added two plots that show the

---

spatial distribution of dissipation and entropy production. The spatial distribution for the local dissipation is now shown in Fig. 3 for the first system, and the spatial distribution for the local entropy production is now plotted in Fig. 4(c) for the second system.

In addition, we provide the following statement in the discussion of the results for the first system: "We show in Fig. 3 the spatial distribution of local dissipation throughout the system for a bin width L = 4.6. In line with the probability distributions for $\Omega(L,t)$ shown in Fig. 2, we observe that local dissipation reaches a maximum when the channel is the widest, *i.e.* where the number of particles in the bin, that contributes to the local current, is the largest. Then, as the channel becomes narrower, local dissipation decreases as the bin contains fewer particles and the local current decreases. Fig. 3 thus shows that our approach enables the mapping of local dissipation in a fluid flowing through a complex environment." and the following statement for the second system:"Our approach thus allows for the mapping of how entropy is produced locally as shown in Fig. 4(c) with an arbitrarily high resolution.".

- *Similarly, the bin definition is important to the analysis; the authors should explain how bins were chosen and justify the bin width of 4.6.*

Author reply: We thank the reviewer for drawing our attention to this point. We have clarified in the caption

---

of Fig. 2 and in the text that we vary systematically the bin width between L = 2 and L = 50.6 to show how localization impacts local dissipation. This is measured by the correlation factor $\kappa(L)$ (shown in the right panel of Fig. 2 for ~20 different values with 2 < L < 50.6) and the slope of the asymetry function (shown for L = 2 and L = 10.1, in addition to the distributions for local dissipation shown for L = 4.6 in the left panel of Fig. 2. Fig. 2 thus reveals that the same behavior is observed for ~20 different L values with 2 < L < 50.6. We chose to show the distributions for a bin width L = 4.6, which corresponds to roughly 10 % of the entire system, as a typical example. We made the following changes:

In the caption of Fig. 2, we added the following in blue: "Local dissipation in bins of length L, with 2 < L < 50.6, for a fluid driven through a channel of varying width. (Left) Local dissipation $\Omega_{L,t}$ over bins of length L = 4.6 and t = 5. The central bin (in black), where the channel is narrower, appears on the left, and is associated with a greater number of trajectories with $\Omega_{L,t} < 0$ than for adjacent bins (in red) and the remaining bins. (Top Right) L-dependence of the correlation coefficient $\kappa_{L,t}$ for ~ 20 different L values with 2 < L < 50.6. (Bottom Right) Test of the local fluctuation theorem. The plot shows that the simulation data for the left-hand-side of Eq. 2 (open diamonds for L = 2) and open squares for L = 10.1) fall onto lines of increasing slope as L decreases."

In the main text, we added the following: "Turning to the analysis of local dissipation in Fig. 2, we examine how localization impacts local dissipation and the local form of the fluctuation theorem. First, we comment on the results obtained for a bin size of L = 4.6, which corresponds roughly to 10% of the entire system," (..) "The top right panel shows the variation of $\kappa_{L,t}$ in the central region, obtained using Eq. 3, as a function of L for 2 < L < 50.6"(..)"We finally examine how localization impacts the ratio of time-reversed probabilities or, in other words, the ratio of the probabilities to observe either a positive or a negative local dissipation. The simulation results, shown for different L in the bottom right panel of Fig. 2, follow the linear relation predicted by the local fluctuation theorem in Eq. 2."

- *The examples are highly idealized (fluids of repulsive disks). While they provide conceptual clarity, it is not obvious how these correspond to experimental active matter systems. If the paper's claim is to address 'complex and active fluids,' the authors should articulate which experimental phenomena these models are meant to capture and why the present framework provides unique insight into them.*

Author reply: We thank the reviewer for raising this point. The systems studied in our work capture key

aspects of flows in complex environments and active systems. Living systems often have to navigate through complex environments in biological settings [31–33] such as, *e.g.*, channels of varying width as modeled in the first system examined in this work or past large obstacles as modeled in the second system we study. Furthermore, several features exhibited by active systems, including the well-known motility-induced phase separation [34] or stimulus-driven pattern formation arising from collective effects in bacteria [35], are captured by models consisting of soft repulsive disks supplemented by an active term,

such as the active Ornstein-Uhlenbeck process model used in the first system studied in our work, as shown by Marchetti *et al.* [36], Hagan *et al.* [37], and others [38–40].

We include the following statement in the conclusion: The systems studied in our work capture key aspects of flows in complex environments and active systems. Living systems often have to navigate through complex environments in biological settings [31–33] such as, *e.g.*, for a fluid flowing in channels of varying width as modeled in the first system examined in this work or a fluid flowing past a large obstacle as modeled in the second system we study. The third system we examine retains key ingredients of minimal active particle models [36–40], which account for motility-induced phase separation [34] or stimulus-driven pattern formation in bacteria [35], by determining local dissipation in a fluid of active particles modeled as disk-shaped repulsive cores supplemented with active Ornstein-Uhlenbeck processes [38, 39].

- *The authors also conclude with "Future work will focus on applying this framework to unravel the onset of complex collective behaviors in active matter." Since the title emphasizes active matter, it would be helpful if the authors made clearer which aspects of experimental active matter their model systems are intended to represent.*

Author reply: We thank the reviewer for this excellent suggestion and include a discussion of how the

---

models and framework introduced here allow for the study of two collective phenomena that only arise in active systems, *i.e.*, motility-induced phase separation [34, 41] and stimulus-driven pattern formation in bacteria [35]. As discussed in the previous point, the minimal model with active Ornstein-Uhlenbeck processes can model these phenomena in active matter [38, 39]. We have revised the conclusion to include the following statement.

Future work will focus on applying this framework to unravel the onset of complex collective behaviors in active matter, most notably to the analysis of motility-induced phase separation [34, 41] and stimulus-driven pattern formation in bacteria [35].

Overall, the framework is rigorous and the results are interesting, but the manuscript in its current form does not yet rise to the level of clarity of a Letter. I encourage the authors either to strengthen the main text (by clarifying claims, explicitly mapping spatial dissipation, and contextualizing their results relative to experimental active matter), or to consider expanding the manuscript into a regular article where more methodological and numerical details can be developed.

Author reply: We thank the reviewer for their positive comments and believe we have addressed all the

---

issues raised by the reviewer. With the implemented changes, we have been able to strengthen the letter by better articulating our claims. In addition, we have added new graphs explicitly mapping the spatial distribution of dissipation, and provided a detailed discussion of the relevance of the systems studied in this work (fluids driven through

narrowing channels or past obstacles, as well as the Active Ornstein-Uhlenbeck Particle (AOUP) model) to experimental active matter.

Bibliography

[1] I. Prigogine, Science 201, 777 (1978).

[2] D. Ruelle, Phys. A 263, 540 (1999).

[3] G. Gallavotti and E. G. D. Cohen, J. Stat. Phys. 80, 931 (1995).

[4] R. Van Zon and E. G. D. Cohen, Phys. Rev. Lett. 91, 110601 (2003).

[5] D. J. Evans, E. G. D. Cohen, and G. P. Morriss, Phys. Rev. Lett. 71, 2401 (1993).

[6] D. J. Evans and D. J. Searles, Adv. Phys. 51, 1529 (2002).

[7] D. J. Evans, D. J. Searles, and S. R. Williams, J. Chem. Phys. 128, 014504 (2008).

[8] D. J. Searles, L. Rondoni, and D. J. Evans, J. Stat. Phys. 128, 1337 (2007).

[9] G. Wang, E. M. Sevick, E. Mittag, D. J. Searles, and D. J. Evans, Phys. Rev. Lett. 89, 050601 (2002).

[10] D. Carberry, J. C. Reid, G. Wang, E. M. Sevick, D. J. Searles, and D. J. Evans, Phys. Rev. Lett. 92, 140601 (2004).

[11] T. M. Squires and T. G. Mason, Ann. Rev. Fluid Mech. 42, 413 (2010).

[12] T. A. Waigh, Rep. Prog. Phys. 79, 074601 (2016).

[13] D. Collin, F. Ritort, C. Jarzynski, S. B. Smith, I. Tinoco Jr, and C. Bustamante, Nature 437, 231 (2005).

[14] K. Hayashi, H. Ueno, R. Iino, and H. Noji, Phys. Rev. Lett. 104, 218103 (2010).

[15] S. Ciliberto, S. Joubaud, and A. Petrosyan, J. Stat. Mech.: Theor. Exp. 2010, P12003 (2010).

[16] É. Fodor, W. W. Ahmed, M. Almonacid, M. Bussonnier, N. S. Gov, M.-H. Verlhac, T. Betz, P. Visco, and F. van Wijland, Europhys. Lett. 116, 30008 (2016).

[17] F. S. Gnesotto, F. Mura, J. Gladrow, and C. P. Broedersz, Rep. Prog. Phys. 81, 066601 (2018).

[18] G. Michel and D. J. Searles, Phys. Rev. Lett. 110, 260602 (2013).

[19] C. Battle, C. P. Broedersz, N. Fakhri, V. F. Geyer, J. Howard, C. F. Schmidt, and F. C. MacKintosh, Science 352, 604 (2016).

[20] J. Li, J. M. Horowitz, T. R. Gingrich, and N. Fakhri, Nat. Commun. 10, 1666 (2019).

[21] I. A. Martínez, G. Bisker, J. M. Horowitz, and J. M. Parrondo, Nat. Commun. 10, 3542 (2019).

[22] A. Ghosal and J. R. Green, Phys. Rev. Res. 7, L012078 (2025).

[23] D. J. Skinner and J. Dunkel, Proc. Natl. Acad. Sci. 118, e2024300118 (2021).

[24] N. M. Boffi and E. Vanden-Eijnden, Proc. Natl. Acad. Sci. 121, e2318106121 (2024).

[25] A. Nir, E. Sela, R. Beck, and Y. Bar-Sinai, Proc. Natl. Acad. Sci. 117, 30234 (2020).

[26] P. J. Foster, J. Bae, B. Lemma, J. Zheng, W. Ireland, P. Chandrakar, R. Boros, Z. Dogic, D. J. Needleman, and J. J. Vlassak, Proc. Natl. Acad. Sci. 120, e2207662120 (2023).

[27] D. J. Searles and D. J. Evans, Phys. Rev. E 60, 159 (1999).

[28] G. P. Morriss and C. P. Dettmann, Chaos 8, 321 (1998).

[29] D. Evans and G. Morriss, Nonequilibrium Statistical Mechanics of Liquids (Cambridge University Press, Cambridge, 2008).

[30] S. R. Williams, D. J. Searles, and D. J. Evans, Phys. Rev. E 70, 066113 (2004).

[31] T. V. Phan, R. Morris, M. E. Black, T. K. Do, K.-C. Lin, K. Nagy, J. C. Sturm, J. Bos, and R. H. Austin, Phys. Rev. X 10, 031017 (2020).

[32] B. Hu and Y. Tu, PLoS Comput. Biol. 10, e1003672 (2014).

[33] A. Gosztolai and M. Barahona, Commun. Phys. 3, 47 (2020).

[34] M. C. Marchetti, J.-F. Joanny, S. Ramaswamy, T. B. Liverpool, J. Prost, M. Rao, and R. A. Simha, Rev. Mod. Phys. 85, 1143 (2013).

[35] J. Arlt, V. A. Martinez, A. Dawson, T. Pilizota, and W. C. Poon, Nat. Commun. 9, 768 (2018).

[36] Y. Fily and M. C. Marchetti, Phys. Rev. Lett. 108, 235702 (2012).

[37] G. S. Redner, C. G. Wagner, A. Baskaran, and M. F. Hagan, Phys. Rev. Lett. 117, 148002 (2016).

[38] D. Martin, J. O'Byrne, M. E. Cates, É. Fodor, C. Nardini, J. Tailleur, and F. Van Wijland, Phys. Rev. E 103, 032607 (2021).

[39] G. P. Nguyen, R. Wittmann, and H. Löwen, J. Phys. Condens. Matt. 34, 035101 (2021).

[40] C. Desgranges, M. Ferrari, P. M. Chaikin, S. Sacanna, M. E. Tuckerman, and J. Delhommelle, Soft Matter 19, 7334 (2023).

[41] S. Ro, B. Guo, A. Shih, T. V. Phan, R. H. Austin, D. Levine, P. M. Chaikin, and S. Martiniani, Phys. Rev. Lett. 129, 220601 (2022).
